# Analysis of Epidemiological Factors and SNP rs3804100 of *TLR2* for COVID-19 in a Cohort of Professionals Who Worked in the First Pandemic Wave in Belém-PA, Brazil

**DOI:** 10.3390/genes14101907

**Published:** 2023-10-05

**Authors:** Marcos Jessé Abrahão Silva, Caroliny Soares Silva, Rebecca Lobato Marinho, Jeanne Gonçalves Cabral, Ellen Polyana da Costa Gurrão, Pabllo Antonny Silva dos Santos, Samir Mansour Moraes Casseb, Karla Valéria Batista Lima, Luana Nepomuceno Gondim Costa Lima

**Affiliations:** 1 Master Program in Epidemiology and Health Surveillance (PPGEVS), Evandro Chagas Institute (IEC), Ananindeua 67030-000, PA, Brazil; 2 Master and PhD Program in Parasitic Biology in the Amazon (PPGBPA), University of State of Pará (UEPA), Belém 66087-670, PA, Brazil; karolinysoares2303@gmail.com (C.S.S.); antonnypabllo@gmail.com (P.A.S.d.S.); 3 Evandro Chagas Institute (IEC), Ananindeua 67030-000, PA, Brazil; rebeccamarinho28@gmail.com (R.L.M.); jeannegoncalves@iec.gov.br (J.G.C.); ellengurrao@iec.gov.br (E.P.d.C.G.); samircasseb@ufpa.br (S.M.M.C.); karlalima@iec.gov.br (K.V.B.L.)

**Keywords:** *TLR2*, single nucleotide polymorphism, COVID-19, epidemiology

## Abstract

COVID-19 is an infectious disease caused by coronavirus 2 of the severe acute syndrome (SARS-CoV-2). Single nucleotide polymorphisms (SNPs) in genes, such as *TLR2*, responsible for an effective human immune response, can change the course of infection. The objective of this article was to verify associations between epidemiological factors and *TLR2* SNP rs3804100 (Thymine [T] > Cytosine [C]) in professionals from Health Institutions (HI) who worked during the first pandemic wave and COVID-19. A case-control study was conducted with Belém-PA HI workers (Northern Brazil), divided into symptomatology groups (Asymptomatic-AS; *n* = 91; and Symptomatic-SI; *n* = 123); and severity groups classified by Chest Computerized Tomography data (symptomatic with pulmonary involvement—SCP; *n* = 35; symptomatic without pulmonary involvement—SSP; *n* = 8). Genotyping was performed by Sanger sequencing, and Statistical Analysis was conducted through the SPSS program. Bioinformatics servers predicted the biological functions of the *TLR2* SNP. There were associations between the presence of comorbidities and poor prognosis of COVID-19 (especially between symptomatology and severity of COVID-19 and overweight and obesity) and between the sickness in family members and kinship (related to blood relatives). The homozygous recessive (C/C) genotype was not found, and the frequency of the mutant allele (C) was less than 10% in the cohort. No significant associations were found for this SNP in this cohort. The presence of SNP was indicated to be benign and causes a decrease in the stability of the TLR2 protein. These data can help the scientific community and medicine find new forms of COVID-19 containment.

## 1. Introduction

Coronavirus disease 2019 (COVID-19) is an infectious disease caused by severe acute respiratory syndrome coronavirus 2 (SARS-CoV-2), a species of beta-coronavirus. Zoonotic-originating disease (probably due to bat overflow) in most cases typically causes signs and symptoms similar to those of influenza, and in about 10 to 20% of cases, it can lead to pneumonia through acute respiratory distress syndrome (ARDS) for 8 to 14 days with dyspnea and reaching hypoxemia, in addition to being asymptomatic [1].

Due to its high transmissibility (through expelled respiratory droplets, aerosols, surface contamination, and fomites), the disease spread rapidly throughout the world [2]. In this context, the World Health Organization (WHO) classified it as a pandemic and public health emergency of international concern just three months after the identification of the first case [3]. After the notification of the pandemic, more than 500 million confirmed cases and about 6 million deaths were registered worldwide, with two waves that had already been notified in several countries, such as Brazil, in some cases, a third wave and a possible fourth wave were announced due to the new variants of the virus [4]. Thus, the number of cases of COVID-19 continues to rise.

Brazil is the second country with the highest number of deaths from the disease [4]. According to epidemiologists, the first wave of COVID-19 in Brazil began on 25 February 2020 and ended around 31 October 2020 [5,6]. In particular, in Brazil, the first wave of the pandemic was characterized by the difficulty in detecting asymptomatic individuals and the deficient policy of availability of diagnostic tests in the country with respect to mass tests for COVID-19, due to the lack of central political leadership in the provision of health networks in the planning and promotion of their management [7,8]. 

Reference diagnosis is based on reverse transcription polymerase chain reaction (RT-PCR), and the association between laboratory and tomographic findings by Chest Computed Tomography (CCT) may be present, even in initial symptomatic patients [9,10,11]. In the first wave of the pandemic, due to the fragile system of availability of confirmatory tests, the considerable increase in cases, and the proximity of disease symptoms to flu syndromes, the WHO recommended that people who had at least two of the suggestive symptoms should already be classified as cases of the disease, which was also adopted by the Brazilian Ministry of Health [12,13]. 

Different clinical manifestations and evolutions of COVID-19 can be related to several factors, from the viral amount to which the individual is exposed (viral load) to sociodemographic, behavioral, immunological, and genetic aspects of the host [14]. The immune system plays a key role in the fight against Severe Acute Respiratory Syndrome—SARS [15]. In fact, the innate immune system is capable of recognizing the molecular structures produced by SARS-CoV-2 infection (by virus invasion) [16].

From this perspective, *Toll-like Receptors* (*TLRs*) are genes discovered, initially in the 1980s in flies of the genus Drosophila and later in mammals, in which they encode transmembrane proteins, characterized by the presence of an extracellular domain N-terminal rich in leucine repeats (LRR) and acting as pattern recognition receptors (PRRs) for the development and activation of the innate immune system by recognizing pathogen-associated molecular patterns (PAMPs) of invading agents. Each TLR can recognize specific types of PAMPs and/or Damage-Associated Molecular Patterns (DAMPs) [17]. 

In the case of SARS-CoV-2 infection, TLR2 can track beta (β)-coronavirus infection through recognition of the E protein, inducing the release of pro-inflammatory cytokines such as TNF-α and interferon-gamma (IFN-γ) [18]. Therefore, there is significant cytokine production in the body and a contribution to the generation of adaptive immunity by monitoring the expression of costimulatory molecules for defense against pathogens, as in the case of SARS-CoV-2 [19]. 

The immunogenetic aspects of the host in relation to infection can be investigated, for example, through single nucleotide polymorphisms (SNPs), which encode an amino acid alteration in genes responsible for an effective human immune response, thus altering the course of viral infection [20]. It is necessary to understand the importance of host immunogenetic heterogeneity in the *TLR2* gene for the evolution of COVID-19 to create strategies to cope with the disease. *TLRs* play a key role in generating and maintaining the innate immune response, in addition to guiding the adaptive immune response in infections. Therefore, SNPs in these genes can generate different levels of receptor expression, generating uncoordinated immune responses through excessive or reduced cytokine production [21,22,23].

Tuberculosis (TB), an essentially lung disease, was the disease most studied for the SNP rs3804100 in the *TLR2* gene in different populations [21]. This may be linked to the fact that the highlighted gene is the main receptor for lipoproteins in mammals, derived from a variety of bacteria, such as the agent *Mycobacterium tuberculosis*. In addition, TLR2 is a crucial factor in activating IFN-γ, which promotes cellular immunity in the Th1 population through the induction of IL-12 [24,25,26]. The main point of similarity between tuberculosis and COVID-19 is the fact that both affect the respiratory system of the infected person. In this context, it is scientifically valid to evaluate the same SNP and its associations for COVID-19.

Therefore, in this study, we propose an evaluation of epidemiological factors and SNP rs3804100 (Thymine to Cytosine, T to C) in the *TLR2* gene of Health Institutions professionals in the city of Belém (capital of the State of Pará, Brazil) who worked during the first pandemic wave to analyze the relationship with symptomatology and clinical development of COVID-19. The use of the first wave of COVID-19 in this work is due to the fact that there were still no variants of the virus that could resort to biases in the analysis of the evolution of the condition of these individuals, as well as the fact that the genetic data analyzed of these individuals reflect the observation that genetic characteristics, such as an SNP of the *TLR2* gene, do not change in an individual during his or her course of life [27].

## 2. Material and Methods

### 2.1. Study Design and Ethical Considerations

This study is characterized as observational with a quantitative analytical nature and is classified as a retrospective case-control. It followed the recommendations of Strengthening the reporting of observational studies in epidemiology (STROBE) [28]. This study protocol was approved by local ethics committees, and all subjects gave their written informed consent (Term of Free and Informed Consent—TCLE). This work was approved by the Research Ethics Committee of the State University of Pará—UEPA (CAAE: 38113620.1.0000.5174) and is related to the research project “Análise da resposta ao SARS-CoV-2 em relação aos achados radiológicos e/ou à susceptibilidade genética individual”, with opinion number: 6.124.862. This research was carried out in accordance with the Helsinki Declaration [29] and Resolution N^o^. 466/2012 of the Brazilian National Health Council [30].

### 2.2. Settings and Participants

This study was carried out in 10 health institutions located in the city of Belém-PA, Brazil (in the Amazon Region—Northern Brazil). They were: Policlínica Metropolitana de Belém (PMB); Jean Bittar Hospital; Hospital das Clínicas Gaspar Vianna; João de Barros Barreto University Hospital; Hospital Adventista de Belém; Dom Vicente Zico Hospital; Women’s Health Hospital; Institute of Hematology and Hemotherapy of Belém (IHEBE); Psychosocial Care Center (CAPS); Secretariat of Public Health (SESPA). Institutions were chosen randomly based on obtaining contacts that fit this study population. All participating institutions received subjects infected with COVID-19 during the period of the first wave of the pandemic. 

This study included, through a convenience sampling, 214 health, administration, and general services professionals who actively worked in health institutions that received individuals with COVID-19 in the period between 1 April 2021 and 30 June 2020, exposed directly and daily to SARS-CoV-2, since during this period all individuals who worked in health institutions can be considered directly exposed to SARS-CoV-2 [31]. During this period, all people who worked in health institutions can be considered exposed to SARS-CoV-2 since safety protocols were not yet well established, there was overcrowding in health institutions, masks were scarce, and the N95 mask was not used [32].

### 2.3. Variables and Division of Cohort in Groups of This Study

These professionals were first divided into two groups. Group 1, which is made up of individuals who were in constant contact with a patient with COVID-19 throughout the first wave but did not report symptoms of COVID-19, was called asymptomatic (AS), and group 2, composed of professionals who were also in direct contact and reported at least two characteristic symptoms of COVID-19 (with or without COVID-19 testing), was designated as symptomatic (SI).

The presentation of two of the main symptoms suggestive of COVID-19 infection and that these symptoms were related to the characteristic clinical presentation of the first wave of COVID-19 (dyspnea, fever, or dry cough) was considered a symptomatological illness criterion [33,34]. 

The second division, from the perspective of aggravation, is composed of groups 3 and 4, which are individuals from group 2, who performed Chest Computerized Tomography (CCT). Group 3 consisted of individuals who reported at least two symptoms and also reported pulmonary impairment ≥ 10%, named symptomatic with pulmonary involvement (called SCP), and group 4 also reported at least two symptoms of COVID-19 and had a CCT with a result without lung injury, this group was named symptomatic without pulmonary involvement (called SSP). The evaluation of severity by CCT in COVID-19 is what was recommended by the Ministry of Health and WHO [35,36].

**Figure 1:**
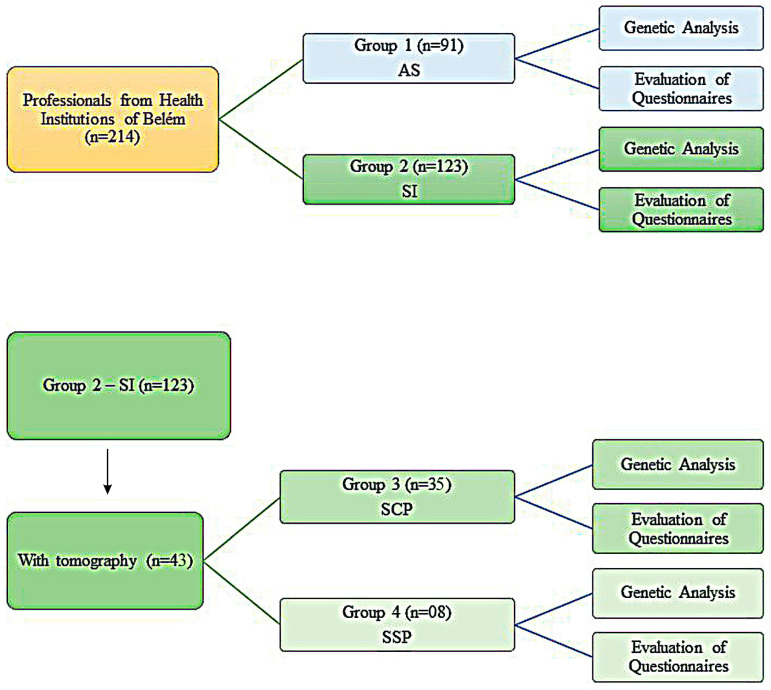
Sample Processing and Classification Flowchart.

Questionnaires developed by this study group were evaluated for epidemiological analysis and possible correlation with the genetic data of these individuals. Information such as signs and symptoms related to the period of the first wave, sex, age, occupation, and comorbidities was used. CCT data were added based on the patient’s self-report while completing the questionnaire. These data were collected from 1 November 2021 to 1 November 2022. Professionals in health, administrative, and general service areas who did not work from April to June 2020 in places that directly assisted patients with COVID-19 or individuals who did not accept to participate in the research or who did not sign the consent form were excluded from the survey. The sampling and its appropriate groups are represented in Figure 1.

### 2.4. Sample Collection, DNA Isolation, and Amplification of the Samples by Polymerase Chain Reaction (PCR)

Sample collection was carried out between 1 June 2021 and 30 March 2022. Blood samples were taken from professionals who got sick and who did not get sick by venipuncture in 5 mL EDTA tubes, stored at −20 °C for later laboratory procedures in the Molecular Biology Laboratory—LABIMOL, Bacteriology and Mycology Section (SABMI) of the Evandro Chagas Institute (IEC). DNA extractions were performed using the DNeasy Blood and Tissue Kit (QIAGEN^®^, Venlo, The Netherlands), following the manufacturer’s instructions. For all these professionals, the SNP rs3804100 of *TLR2* was evaluated to correlate with the susceptibility and severity of COVID-19. The SNP information (SNP ID) of *TLR2* was retrieved from the National Center for Biotechnology Information—NCBI, dbSNP (http://www.ncbi.nlm.nih.gov/snp/; accessed on 1 January 2022) [37].

The typification of the SNP of the *TLR2* gene was carried out by sequencing through amplification of the DNA by the nucleotide primers (primers) for the Polymerase Chain Reaction—PCR, which were designed by the Primer3Plus program version 2.0 (http://www.bioinformatics.nl/primer3plus/; accessed on 10 December 2021) [38] from the respective genomic regions deposited in GenBank [39]. Thus, 1 primer (with Forward Strand—ACCGGAGAGACTTTGCTCAC and Reverse Strand—GCTTGCTGCTCCTGAGTGAA, 437 base pairs) at the 5′ Binding site 153704120 and reference NC_000004.12 were designed for use and amplification [37]. They were performed with Platinum Taq DNA Polymerase, DNA-free (Invitrogen^®^, Thermo Fisher Scientific Corporation, Waltham, MA, USA) using the following conditions in a thermocycler: initial denaturation at 95 °C for 1 min, followed by 35 cycles of denaturation at 95 °C to 30 s, annealing at the appropriate temperature for this primer (65 °C) for 30 s, extension at 72 °C to 1 min, and after that, final extension at 72 °C for 10 min [40]. The amplified products were subjected to electrophoresis in a 2% agarose gel containing 3.0 µL of Sybr Safe (Invitrogen^®^, Thermo Fisher Scientific Corporation, Waltham, MA, USA) to visualize the amplified DNA fragments in a photodocumenting device. 

### 2.5. Running Samples in Capillary Electrophoresis

Purification of PCR products was performed using the EasyPure PCR Purification Kit (TransGen Biotech Co.^®^, Beijing, China), according to the manufacturer’s recommendations. The already purified amplified products were submitted to the sequencing reaction following the instructions of the BigDye X-Terminator kit to the ABI 3130 Genetic Analyzer sequencer (Applied Biosystems^®^, Life Technologies, Thermo Fisher Scientific Corporation, Waltham, MA, USA) for visualization and analysis of the areas of interest of SNP through the Bioedit program version 7.2.5 [41], respectively, with subsequent performance of BLAST on the NCBI website (https://blast.ncbi.nlm.nih.gov/Blast.cgi/; accessed on 15 August 2023).

### 2.6. Presentation of Data and Statistical Analysis of Results

Information regarding laboratory results was organized in a database using the Microsoft Office Access program (Microsoft Corp^®^, Redmond, WA, USA) and presented through graphs or tables generated by the Microsoft Office Excel program (Microsoft Corp.^®^, Redmond, WA, USA). The observed proportions of the presence of the SNP within each studied group were analyzed with the aid of IBM SPSS Statistics v. 26.0 software (IBM Corp.^®^, Armonk, NY, USA), using the G, two-tailed chi-square (χ^2^), and Fisher’s exact tests to verify the association between variables arranged in 2 × 2 tables. An Odds Ratio (OR) test with a 95% confidence interval (CI) was used to assess the association between exposure and the outcomes of interest. A probability (*p*) ≤ 0.05 was considered statistically significant. 

Genotype frequencies were tested for the Hardy-Weinberg Equilibrium (HWE) using the chi-square (χ^2^) test with *p* < 0.001 as the cut-off point for the significance level through Arlequin version 3.5.1.2 [42,43]. The G*Power software version 3.1.9.7 was used to determine the power of the sample size using a goodness-of-fit Chi-squared test [44]. In addition to that, the biological functions of the SNP were evaluated by PolyPhen-2 (Polymorphism Phenotyping, http://genetics.bwh.harvard.edu/pph2/; accessed on 22 January 2023) through the UniProt Database Entry O60603 (Human TLR2) [45], while protein structural stability was evaluated using I-Mutant 2.0 with Protein Data Bank—PDB Code 2Z7X (https://folding.biofold.org/i-mutant/i-mutant2.0.html/; accessed on 27 March 2023) [46].

## 3. Results

### 3.1. Power of Sample Size, Normality of Variables, and Hardy-Weinberg Equilibrium (HWE)

The power of the sample size was estimated using Chi-square quality-of-fit for symptomatic (N = 123) and asymptomatic individuals with COVID-19 (N = 91), with an α error probability of 0.05 and an effect size of 0.3. The actual real power (1-β error probability) was 0.99, which is greater than 0.80, i.e., statistically acceptable [44]. The variables analyzed in this study were disposed of in a categorical and nonparametric way, characterized by an absolute count and percentage. The SNP rs3804100 was in agreement with the HWE (*p* = 0.93).

### 3.2. Baseline Characteristics Associated with COVID-19 Symptomatology among Individuals in the Belém Professional Cohort

The epidemiological characteristics and comorbidities associated with the cohort are presented in Table 1. There were no statistically significant differences between the age group and sex categories and COVID-19 (*p* > 0.05). However, with respect to pre-existing comorbidities in these subjects, the absence of comorbidities was associated with the group of asymptomatic individuals, in which the absence of comorbidity is associated with a two times greater chance of being asymptomatic (*p* = 0.0034; OR = 2.61 [95% CI = 1.35–5.01]). The number of comorbidities was also a significant factor for symptomatology in the individuals, and, therefore, the presentation of two or more comorbidities was statistically significant and was present only in the symptomatic group (*p* = 0.020; OR = 0.66 [95% CI = 0.54–0.81]). The types of comorbidities are shown in Table 1. Among these, the presence of Diabetes Mellitus or Overweight and obesity were associated with the group of people with symptoms of COVID-19 (*p* = 0.021, OR= 0.56 [95% CI = 0.49–0.63]; *p* = 0.003, OR = 4.17 [95% CI = 1.52–11.40], respectively). 

Table 2 shows the analysis between the AS and SI groups and the illnesses of their family members. Thus, it was found that blood relatives who live with professionals had 2.33 higher chances of becoming ill (*p* = 0.016; OR = 2.33 [95% CI = 1.16–4.69]).

### 3.3. Baseline Characteristics Associated with the Severity of COVID-19 among Individuals in the Cohort of Professionals from Belém

The epidemiological characteristics and associated comorbidities were also analyzed in relation to the severity of the disease (between the AS, SCP, and SSP groups), according to the tomographic results of the subjects, and are displayed in Table 3. 

Comparison between the AS and SCP groups showed that people with pre-existing comorbidities are 8 times more likely to have lung injuries (*p* < 0.001; OR = 7.9 [95% CI = 3.31–18.98]). Analysis between the SCP and SSP groups demonstrated a 10-fold increase in the risk (*p* = 0.016; OR = 10.26 [95% CI = 1.14–92.25]) of having pulmonary involvement in symptomatic individuals with pre-existing comorbidities. In the comparison between the AS and SCP groups, no asymptomatic individual had two or more comorbidities (*p*= 0.012; OR = 0.48 [95% CI = 0.33 = 0.69]).

**Figure 2:**
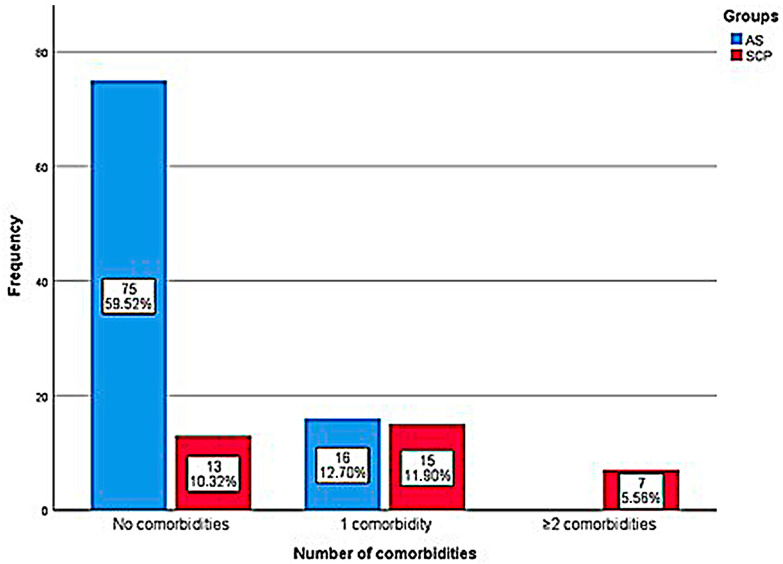
Graphic representation in bars of the absolute and relative number of pre-existing comorbidities in relation to the gravity between the groups of AS and SCP individuals in the cohort.

The distribution and proportion of the number of individuals with or without comorbidities and lung injury in the AS and SCP groups is shown in Figure 2, which reports that symptoms and lung damage are present only in those with 2 or more comorbidities (100%). In these groups (AS and SCP), the analysis found that asthma (*p* = 0.018; OR = 4.5 [95% CI = 1.18–17.06]), cardiopathies (*p* = 0.022; OR = 0.26 [95% CI = 0.19–0.35]), Diabetes Mellitus (*p* = 0.001; OR = 0.25 [95% CI = 0.18–0.34]), Systemic Arterial Hypertension—SAH (*p* = 0.038; OR = 3.55 [95% CI = 1.01–12.53]), and overweight and obesity (*p* < 0.001; OR = 8.97 [CI 95% = 2.86–28.06]) were significantly correlated; however, for the other investigations between the AS and SSP and SCP and SSP groups and types of comorbidities, no significant associations were observed. 

The distribution of individuals with family members who were sick or not and their relationship with kinship and cohabitation were characterized in Table 4 according to the severity groups of the disease. From a family point of view, there was a significant association between two times higher chances of severe illness in family members of study subjects when comparing the SCP with the AS group (*p* = 0.022; OR = 2.86 [95% CI = 1.13–7.24]). Relatives living with the research participant had about seven times more chances of being part of the SSP group in comparison with being a relative of the AS group (*p* = 0.04; OR = 7.25 [95% CI = 0.81–64.45]). 

### 3.4. Genotyping Data for TLR2 SNP rs3804100 Related to Symptomatology and Severity of COVID-19

**Figure 3:**
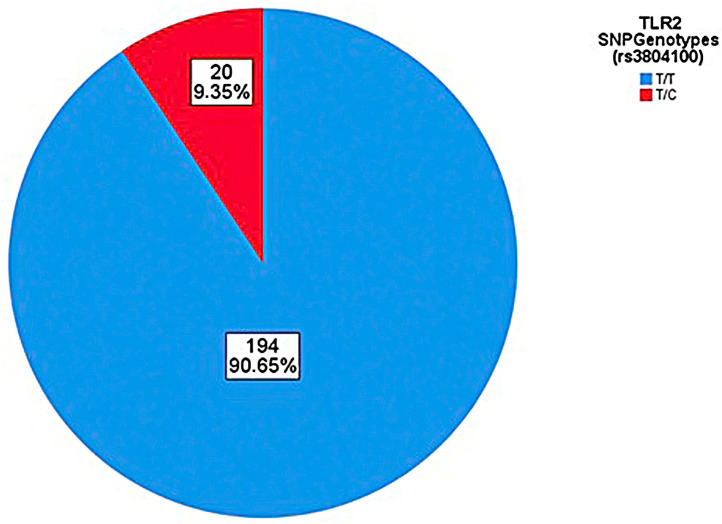
Genotypic distribution of the rs3804100 polymorphism in the sampling.

**Figure 4:**
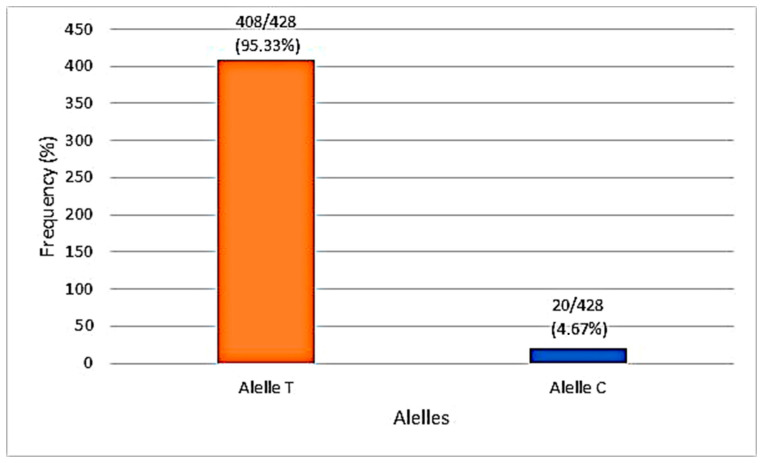
Allelic distribution of the rs3804100 polymorphism in the sampling. Source: Prepared by authors in Microsoft Office Excel 365 (2023).

Genotyping of the *TLR2* SNP rs3804100 is described below, with proportions and associations always being performed by observing the wild-type SNP allele (T) in relation to the mutant (C). For the SNP rs3804100, samples of 214 individuals from the cohort were sequenced, which were analyzed based on the distribution of alleles and genotypes (Figure 3 and Figure 4). No participant in all groups in this research had the C/C genotype (homozygous recessive genotype). Table 5 shows the absolute and relative frequency and associations of the genotypes and alleles found for the SNP rs3804100 in this cohort in relation to the AS and SI groups. 

Table 6 analyzes the absolute and relative frequency of the genotypes and alleles found for the SNP rs3804100 in this cohort in relation to the severity groups of the disease. No participant in the SSP group of this research had the T/C or C/C genotypes.

### 3.5. Analysis of the Characteristics of the Non-Synonymous Mutation rs3804100

Functional instability in proteins can occasionally result from structural alterations in amino acid residues due to variations in protein translation. While I-Mutant 2.0 employs a support vector machine (SVM) for the automated prediction of protein stability changes after mutations, PolyPhen-2 works with the sequence, structural, and phylogenetic information of mutations [47]. 

Using PolyPhen-2, the mutation score, which ranges from 0 to 1, is considered to evaluate if it is harmful. A mutation is considered harmful if the score is close to 1 [48]. I-Mutant 2.0 predicts changes in protein stability (which determines a protein’s conformational form) using free energy change values (ΔΔG), where a negative value of ΔΔG denotes a protein’s declining stability and ΔΔG > 0 suggests an increase in stability in the protein’s structural conformation. Misfolding, protein breakdown, or abnormal protein aggregation can all result from changes in protein stability [49]. 

**Figure 5:**
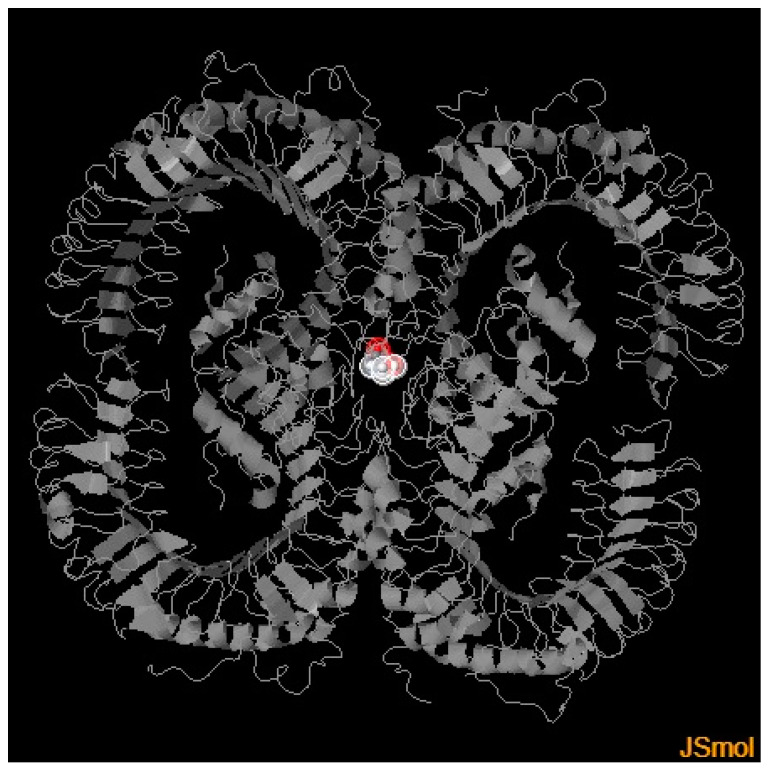
Three-dimensional model of the human TLR2 protein with colored marking in the amino acid change region (Ser450Arg) in its structure due to the presence of the SNP rs3804100. Source: Generated by the PolyPhen-2 server through the Protein Data Bank (PDB)/DSSP Database with UniProt Database Entry O60603 (Human TLR2) and Entry ID 6NIG.

PolyPhen-2 results demonstrated a Benign effect (most likely lacking any phenotypic effect on protein structure or function) prediction for the presence of a mutation in the *TLR2* gene, with a score of 0.002, while I-Mutant 2.0 showed a decreased stability to the protein, with a ΔΔG of −1.35. The 3D model generated by PolyPhen-2 for visualization of structural alterations by the *TLR2* SNP rs3804100 is shown in Figure 5.

## 4. Discussion

Risk factors are conditions that increase the likelihood of becoming ill due to a health problem or infectious disease. The most important host risk factors for COVID-19 infection are age, male gender, and comorbid chronic conditions such as hypertension, type 2 diabetes mellitus, obesity, etc. [50]. CCT can be used in symptomatic patients, even with reports of greater sensitivity; however, with limited specificity in relation to virus detection by the standard molecular test [10]. Regarding the use of tomographic results to assess the severity of the disease, this division was chosen to verify whether the group considered more severe had more significant changes, and the SCP group, due to presenting pulmonary involvement greater than or equal to 10%, was considered the most serious parameter [51]. 

Although it was not possible to characterize bilateral pneumonia with ground-glass manifestation in CCT in these individuals, this analysis of the severity of the disease was carried out in conjunction with the investigation of symptoms suggestive of COVID-19 (particularly to the first pandemic wave) in study professionals directly exposed to the virus. This perspective was considered for the methodology, taking into account the Brazilian reality at the time of scarcity of diagnostic tests and the low availability of individual and collective protective equipment (EPIs/EPCs) in health services (a situation that was still more pronounced in regions far from large urban centers, such as the Amazon Region), and it reduces the risk of bias in relation to the diagnostic question of the research subjects [52,53,54].

In the present case-control study, the age group did not have a significant relationship between any analyses of the groups involved; however, it must be taken into account that the sample had a work requirement, with most individuals in the 19–50 age category, which represents a percentage of 91.12% of the total number of participants in the cohort, while the age group over 50 years was only 8.88% of the total, with 8.8% in the asymptomatic group and 8.9% in the symptomatic group. The literature reports that SARS-CoV-2 can infect people of any age; however, it is significantly less prevalent and frequently asymptomatic in children and young adults under the age of 14, and, in the first pandemic wave, before the availability of vaccination, the median age of death of the disease was 75 years [55,56]. 

With regard to gender, no significant statistical associations were observed either, so that all groups had a majority of female members; however, the gender sampling also has an employment relationship, since, in occupational positions in the area of health, the presence of female employees is greater [57]. Nevertheless, the literature indicates a greater risk of severity for men. In an intensive care unit (ICU) cohort from Italy, 82% were men [58]. This disparity in the severity and mortality of COVID-19 may be partially explained by sex-based variations in the expression of the ACE2 receptor and TMPRSS2 [59]. The patient’s profession category did not show significant differences between the groups analyzed, regardless of being in the administrative sector, general services, or professional in the health area.

Comorbidities are observed in 24% to 51% of hospitalized patients but in 68% to 72% of ICU patients, indicating that severe COVID-19 is related to them [60]. Among the comorbidities found in this current cohort, Overweight and obesity, and Diabetes Mellitus were significantly associated with symptomatic individuals. Furthermore, it was observed that in the SCP group, 62.9% (22/35) had at least one comorbidity. The most frequent comorbidities were descending: overweight and obesity (38.16%); systemic arterial hypertension and Asthma (19.74% each); Diabetes mellitus (9.21%); heart diseases (5.26%); glaucoma and autoimmune diseases (2.76% each); kidney disease and pulmonary fibrosis (1.32% each). These data are corroborated by the risk factors already observed for COVID-19 in the literature [60,61].

Numerous endocrine changes, including cortisol insufficiency, hypogonadism, and hypothyroidism, play a role in mediating the negative correlation between obesity and the results of COVID-19 [62]. Due to a possible intensification of the inflammatory response to COVID-19 infection and the resulting changes in T cell-mediated immunity, persistent inflammation in obese individuals is hypothesized to be a contributing factor to the observed higher mortality [63,64,65]. 

Different regions of the world showed varying prevalence rates of COVID-19 among asthmatics. Although early research from China found a low frequency of asthma among COVID-19 patients [66,67,68,69], some US data [70] suggested an increased prevalence of COVID-19 among children with asthma compared to the adult population (14.4% versus 7.8%, respectively), and, in the USA, it is a risk factor for hospitalization [71]. In Europe, there were regional differences in the prevalence of asthma [72,73,74,75,76]. An American study also found that asthma was independently related to a longer intubation time for COVID-19. Obese people with the coexistence of asthma are at much higher risk of a deteriorating illness course from the illness [77].

These variations in epidemiology might be caused by several factors. Various studies use various methods to diagnose asthma, which can lead to overdiagnosis or underdiagnosis. Furthermore, the prevalence of asthma varies by county, which may be the result of variations in environmental exposure, socioeconomic status, and genetic predisposition [78].

The severity and mortality of COVID-19 appear to be correlated with the presence of diabetes mellitus and individual hyperglycemia levels. Poor glycemic control increases the likelihood of SARS-CoV-2 infection in diabetics and increases the death rate, treatment requirements, and hospitalizations [79]. Cardiovascular illnesses such as myocarditis, arrhythmia, cardiogenic shock, heart failure, and other thromboembolic events are caused by the effect of the infection on the cardiovascular system. In addition to hypertensive individuals, the vascular endothelium can activate monocytes, resulting in almost uncontrollable cytokine production that may have a connection to COVID-19 [80]. 

In this present study, it was also possible to verify that, although not statistically significant, in the symptomatic group, 67.48% of the consanguineous relatives of these individuals became ill, and, among these relatives, 69.88% of the household members became ill, with about seven times greater chance of becoming ill compared to the extradomiciliary. These data suggest that the illness of family members was influenced by contact in the same house with health professionals. These data suggest that family illness is linked to an association of genetic and environmental factors. Regarding ambiental aspects, it is known that to prevent the spread of SARS-CoV-2 infected viral particles and aerosols, it is recommended to avoid interpersonal contact, wash your hands after touching any physical surfaces in anthropogenic settings, and adhere strictly to respiratory etiquette rules [81]. When dealing with people who have a suspected or proven infection, quarantine and confinement are also recommended to prevent disease transmission [82,83].

Subjects without associated risk factors also experience symptoms and worsening of the disease, indicating that genetic factors can also affect the condition and that SNPs in specific genes can affect the variation in the incidence and severity of the disease by dysregulating the host immune response. The innate immune response originates from viral entry into the human cell. When entering the body, the first form of contact between SARS-CoV-2 and humans is through the PRRs of antigen-presenting cells (APCs), such as monocytes and dendritic cells (DCs), which recognize the PAMPs and DAMPs of the virus. In this context, the main PRRs responsible for detecting this virus are TLRs, retinoic acid-inducible I-like receptors (RLRs), nucleotide-binding oligomerization domain-like receptors (NLRs), and absent in melanoma 2 (AIM-2) [84,85]. These proteins induce signaling pathways, such as interferon receptor genes alpha (*IFNAR*) *1* and *IFNAR2*, which produce interferon (IFN)-I and IFN-III, mediators already considered potential inhibitors of the virus in vitro [86].

TLR2 is an extracellular sensor present in the membrane of immune cells, such as macrophages and DCs, that, through pairs or dimers, performs its function of detecting the pathogen through the innate immune response. The ability of TLR2 to differentiate between diacyl and triacyl lipopeptides is achieved by homodimerization or heterodimerization with TLR1 and TLR6, respectively [87]. It can recognize a number of bacterial, viral, fungal, and parasitic agents [88]. 

In the case of SARS-CoV-2 infection, in vitro analysis in mice demonstrated that anti-TLR2 therapy has positive impacts on the survival of these animals. Disruption of TLR2 did not affect SARS-CoV-2-mediated type I IFN-α (a type of IFN-I) production but did significantly alter IL-6 production (a marker of a poor prognosis) [18]. A study by Van Der Sluis et al. (2022) confirmed that plasmacytoid DCs (pDCs) produce IL-6 in response to TLR2 and TLR2/6-mediated sensing of SARS-CoV-2 glycoproteins [89]. Furthermore, there is evidence that activation of the NF-kB inflammatory pathway in COVID-19 (a disease aggravating factor) by protein S is dependent on TLR2 signaling [90]. Therefore, the induction of TLR2 by the human innate immune system probably has more unfavorable outcomes than a protective effect in COVID-19. However, this study focuses on specific *TLR2* genes rather than *IFN* or *IL-6* genes because we place an emphasis on studying the analysis of genes that encode immune cell receptors.

The human *TLR2* gene is located on chromosome 4q31.3 [91]. This gene consists of 3 exons (coding regions) [92]. There are several SNPs in the *TLR2* gene, including the SNP rs3804100. The SNP rs3804100, also known as 1350T/C, is a non-synonymous mutation of the missense type; that is, it leads to a change in the codon from an amino acid in Serine (Ser) to Arginine (Arg) at residue 450. This SNP was first described in a study by Butty et al. (2008) in an analysis of their susceptibility to type 1 diabetes, in which this polymorphism did not have significant differences in frequencies between diabetic progressors and non-progressors [93]. 

The frequency of the mutant allele (C) of this SNP found in the present study for the Amazonian population, more precisely Belém city, was 9.35%, a slightly different percentage from that found reported in the NCBI for the Latino population. americana, estimated at 7.52% [37]. An immunogenetic study with the same SNP in the region of Brazil, the State of Pará, but under analysis for patients with leprosy, found a frequency of the mutant allele of approximately 4.1% and also did not find its homozygous recessive genotype (C/C) [94]. 

This SNP was associated with a decrease in exonic splicing site domains, however, with no defined function in *TLR2* gene expression yet [95,96,97]. Therefore, this study proposes an evaluation of the biological and conformational protein effects of this SNP in *TLR2* through computational prediction. The results demonstrated that this SNP is benign in individuals and causes a decrease in the stability of the TLR2 protein, which may indicate an inverse relationship to the original function of TLR2 in a given population with a disease, generated by a possible functional instability in TLR2 that is not yet fully understood. 

The presence of *TLR2* SNPs in each population can functionally affect the production of the receptor and therefore impact the generation of an effective immune response [21]. Despite having been predicted in this present work about the benign nature and the reduction in the protein stability of TLR2 related to the presence of the SNP rs3804100 and the literary scenario more related to the patient’s worse prognosis when this gene is more expressed, in the case of the Amazonian population, significant associations for any investigated COVID-19 phenotype were not observed. This may simply indicate that this gene region has different genetic variability in people from Belém in relation to other populations, due to gene-gene and gene-environment interactions [27].

Understanding the genetic basis of the host refers to a possible correlation with the manifestation of diseases, as genetic expression in DNA is capable of deregulating in certain people the construction of an effective response against possible health problems [98]. So, this present study is a pioneer in the search for associations between this *TLR2* SNP and the severity and manifestations of COVID-19 in the Brazilian population and the second to investigate these associations with the disease worldwide. In addition to that, it sought to predict biological functions and protein relationships related to the presence of this SNP through bioinformatics analysis.

The study by Salamaikina et al. (2022) with a Russian population was the only study to date investigating this SNP and COVID-19; however, it had a different objective from this present study, which was to compare the allele frequencies of genetic polymorphisms in *TLR* genes in samples obtained from individuals with pneumonia in the period before the appearance of SARS-CoV-2 and during the COVID-19 pandemic in this same population and with international public data from the European population. In contrast to the results of this present research, they concluded that Russian individuals with the mutant allele (C) of the SNP rs3804100 have a 0.32-fold greater vulnerability to COVID-19 pneumonia compared to the group of healthy people [99].

The results of the association of SNPs can vary from one population to another due to the background phenomenon that, together with the environmental circumstances of each distinct population group, can determine the immunogenetic heterogeneity [100,101]. In this sense, further research is needed to better visualize epidemiological and genetic parameters and their consequences in COVID-19. Moreover, more studies are required to complement the information obtained, and more SNPs of the *TLR2* gene must be looked at in relation to the clinical manifestations and severity in this group or the same SNP in cohorts with a larger sample number.

Being a retrospective study, one of the drawbacks of this study is that a COVID-19 test could not be conducted in all of the cohorts, as we were unable to reach the patients when they were actively working on the first wave in the health centers. Additionally, during the first wave, many healthcare providers did not perform tests for the disease, as they were hard to come by and in short supply [102]. Since it was not possible to access tomography data for the entire cohort, the dyspnea symptom was excluded from a possible severity classification because it was impossible to define it as a COVID-19 symptom or a psychological factor. Instead, it was only considered at the end of the analyses based on the participants’ reports from the questionnaires. Additionally, it has memory constraints since the data we evaluated was based on a questionnaire. Our research, however, is restricted to the time of the first wave, which we believe individuals may continue to remember since it was such a horrific and significant time in recent history [103,104].

## 5. Conclusions

This work looked for associations between risk factors and the *TLR2* SNP rs3804100 in Health Institution workers and COVID-19. In this way, a relationship was observed between the occurrence of symptoms (SI) and comorbidity, with a higher prevalence of overweight and obese individuals. Similarly, a relationship was established between the occurrence of symptoms and pulmonary involvement and having two or more comorbidities, with a higher prevalence of comorbidities such as diabetes mellitus, overweight and obesity, systemic arterial hypertension, heart disease, and asthma.

The presence of comorbidity was also more present in the group of symptomatic subjects with pulmonary involvement than in those with asymptomatic ones, and it also predominated in those with lung injury to the detriment of those without lung injury. There was an observation that 100% of the symptomatic professionals with pulmonary involvement in the cohort had ≥2 comorbidities.

There was an association between sick blood relatives living in the same household as those with symptoms and a relationship between illness in blood relatives and symptomatology with pulmonary involvement (irrespective of the household). Furthermore, consanguineous relatives of cohort subjects without pulmonary involvement became significantly ill at the same home.

In the genotype analysis of the *TLR2* SNP rs3804100 (−1350 T/C), the homozygous recessive (C/C) genotype was not found, and the frequency of the mutant allele (C) was less than 10% in the cohort. No significant associations were found for this SNP in this cohort of Belém Health Institutions professionals in the analysis for symptomatology or severity. Furthermore, bioinformatics analysis suggested that the presence of this SNP is benign in individuals and causes a decrease in the stability of the TLR2 protein.

Therefore, the observed data on epidemiological and immunogenetic markers can help the scientific community and medicine seek new forms of containment (especially from the point of view of professionals in the clinical-hospital structure) and combat the disease, such as those focused on anti-TLR2 therapy.

## Figures and Tables

**Table 1:** Epidemiological characteristics of professionals exposed to SARS-CoV-2.

Variable *n* (%)	AS (*n* = 91) *n* (%)	SI (*n* = 123) *n* (%)	*p*-Value
**Age group**			
19–34 years	40 (44%)	63 (51.2%)	*p* > 0.05
35–50 years	43 (47.2%)	49 (39.9%)	
>50 years	8 (8.8%)	11 (8.9%)	
**Sex**			
Female	67 (73.6%)	81 (65.9%)	*p* > 0.05
Male	24 (26.4%)	42 (34.1%)	
**Presence of pre-existing comorbidities**			
No comorbidities	75 (82.4%)	79 (64.2%)	***p* = 0.0034 ^a^**
With comorbidities	16 (17.6%)	44 (35.8%)	
**Quantity of comorbidities**			
1 comorbidity	16 (100%)	32 (72.73%)	***p* = 0.020 ^b^**
≥2 comorbidities	0 (0%)	12 (27.27%)	
**Types of comorbidities**			
Asthma	4 (25%)	11 (18.33%)	*p* > 0.05
Cardiopathies	0 (0%)	4 (6.67%)	*p* > 0.05
Diabetes mellitus	0 (0%)	7 (11.67%)	***p* = 0.021 ^c^**
Systemic Arterial Hypertension (SAH)	5 (31.25%)	10 (16.67%)	*p* > 0.05
Overweight and obesity	5 (31.25%)	24 (40%)	***p* = 0.003 ^d^**
Autoimmune disease	2 (12.5%)	0 (0%)	*p* > 0.05
Kidney disease	0 (0%)	1 (1.66%)	*p* > 0.05
Pulmonary fibrosis	0 (0%)	1 (1.66%)	*p* > 0.05
Glaucoma	0 (0%)	2 (3.33%)	*p* > 0.05
**Profession category**			
Administrative	23 (25.3%)	37 (30.1%)	*p* > 0.05
Healthcare professional	56 (61.5%)	65 (52.8%)	
General Services	12 (13.2%)	21 (17.1%)	

Caption: AS = Asymptomatic Group; SI = Symptomatic Group. Note: ^a^ OR = 2.61 (95% CI = 1.35–5.01); ^b^ OR = 0.66 (95% CI = 0.54–0.81); ^c^ OR = 0.56 (95% CI = 0.49–0.63); ^d^ OR = 4.17 (95% CI = 1.52–11.40).

**Table 2:** Kinship and housing relationships of family members of individuals exposed to SARS-CoV-2.

Variable *n* (%)	Kinship	AS (*n* = 91) *n* (%)	SI (*n* = 123) *n* (%)	*p*-Value
**Getting sick**				
Relatives who did not get sick	Blood Relatives	38 (41.76%)	40 (32.52%)	*p* > 0.05
Relatives who became ill		53 (58.24%)	83 (67.48%)	
Living with the research participant		24 (45.28%)	58 (69.88%)	***p* = 0.016 ^a^**
Not living with the research participant		29 (54.72%)	25 (30.12%)	
**Getting sick in the same household**				
Relatives who did not get sick	Non-blood relatives	64 (70.3%)	73 (59.3%)	*p* > 0.05
Relatives who became ill		27 (29.7%)	50 (40.7%)	

Caption: AS = Asymptomatic Group; SI = Symptomatic Group. Note: ^a^ OR = 2.33 (95% CI = 1.16–4.69).

**Table 3:** Epidemiological characteristics of this cohort of professionals exposed to SARS-CoV-2 in the first wave of the pandemic based on COVID-19 severity.

Variable *n* (%)	AS (*n* = 91) *n* (%)	SCP (*n* = 35) *n* (%)	SSP (*n* = 08) *n* (%)	*p*-Value (AS vs. SCP)	*p*-Value (AS vs. SSP)	*p*-Value (SCP vs. SSP)
**Age group**						
19–34 years	40 (44%)	12 (34.3%)	4 (50%)	*p* > 0.05	*p* > 0.05	*p* > 0.05
35–50 years	43 (47.3%)	18 (51.4%)	4 (50%)			
>50 years	8 (8.8%)	5 (14.3%)	0			
**Sex**						
Female	67 (73.6%)	21 (60%)	6 (75%)	*p* > 0.05	*p* > 0.05	*p* > 0.05
Male	24 (26.4%)	14 (40%)	2 (25%)			
**Presence of pre-existing comorbidities**						
No comorbidities	75 (82.4%)	13 (37.1%)	7 (87.5%)	***p* < 0.001 ^a^**	*p* > 0.05	***p* = 0.016 ^b^**
With comorbidities	16 (17.6%)	22 (62.9%)	1 (12.5%)			
**Quantity of comorbidities**						
1 comorbidity	16 (100%)	15 (68.2%)	0	***p* = 0.012 ^c^**	*p* > 0.05 *	*p* > 0.05
≥2 comorbidities	0	7 (31.8%)	1 (100%)			
**Types of comorbidities**						
Asthma	4 (25%)	6 (18.75%)	1 (50%)	***p* = 0.018 ^d^**	*p* > 0.05 *	*p* > 0.05
Cardiopathies	0	2 (6.25%)	0	***p* = 0.022 ^e^**	-	*p* > 0.05
Diabetes mellitus	0	4 (12.5%)	0	***p* = 0.001 ^f^**	-	*p* > 0.05 *
Systemic arterial hypertension	5 (31.25%)	6 (18.75%)	0	***p* = 0.038 ^g^**	*p* > 0.05 *	*p* > 0.05 *
Overweight and obesity	5 (31.25%)	12 (37.5%)	1 (50%)	***p* < 0.001 ^h^**	*p* > 0.05 *	*p* > 0.05
Autoimmune disease	2 (12.5%)	0	0	*p* > 0.05 *	*p* > 0.05 *	-
Pulmonary fibrosis	0	1 (3.125%)	0	*p* > 0.05 *	-	*p* > 0.05 *
Glaucoma	0	1 (3.125%)	0	*p* > 0.05 *	-	*p* > 0.05 *
**Profession category**						
Administrative	23 (25.3%)	6 (17.1%)	2 (25%)	*p* > 0.05	*p* > 0.05	*p* > 0.05
Healthcare professional	56 (61.5%)	25 (71.4%)	6 (75%)			
General Services	12 (13.2%)	4 (11.4%)	0			

Caption: AS = Asymptomatic Group; SCP = Symptomatic Group with Pulmonary Compromise; SSP = Symptomatic Without Pulmonary Compromise. Note: ^a^ OR = 7.9 (95% CI = 3.31–18.98); ^b^ OR = 10.26 (95% CI = 1.14–92.25); ^c^ OR = 0.48 (95% CI = 0.33 = 0.69); ^d^ OR = 4.5 (95% CI = 1.18–17.06); ^e^ OR = 0.26 (95% CI = 0.19–0.35); ^f^ OR = 0.25 (95% CI = 0.18–0.34); ^g^ OR = 3.55 (95% CI = 1.01–12.53); ^h^ OR = 8.97 (95% CI = 2.86–28.06). * Fisher’s Exact Test.

**Table 4:** Kinship and housing relationships of family members of individuals exposed to SARS-CoV-2 according to the COVID-19 severity of this cohort of professionals.

Variable *n* (%)	Kinship	AS (*n* = 91) *n* (%)	SCP (*n* = 35) *n* (%)	SSP (*n* = 08) *n* (%)	*p*-Value (AS vs. SCP)	*p*-Value (AS vs. SSP)	*p*-Value (SCP vs. SSP)
**Getting Sick**							
Relatives who did not get sick	Blood relatives	38 (41.76%)	7 (20%)	1 (12.5%)	***p* = 0.022 ^a^**	*p* > 0.05	*p* > 0.05
Relatives who became ill		53 (58.24%)	28 (80%)	7 (87.5%)			
Living with the research participant		24 (45.28%)	18 (64.29%)	6 (85.71%)	*p* > 0.05	***p* = 0.04 ^b^**	*p* > 0.05
Not living with the research participant		29 (54.72%)	10 (35.71%)	1 (14.29%)			
**Getting sick in the same household**							
Relatives who did not get sick	Non-blood relatives	64 (70.3%)	20 (57.1%)	5 (62.5%)	*p* > 0.05	*p*> 0.05	*p* > 0.05
Relatives who did not get sick		27 (29.7%)	15 (42.9%)	3 (37.5%)			

Caption: AS = Asymptomatic Group; SCP = Symptomatic Group with Pulmonary Compromise; SSP = Symptomatic Without Pulmonary Compromise. Note: ^a^ OR = 2.86 (95% CI = 1.13–7.24); ^b^ OR = 7.25 (95% CI = 0.81–64.45).

**Table 5:** Genotyping of this cohort of professionals for the *TLR2* SNP rs3804100 for symptomatology association.

Genotyping *n* (%)	AS (*n* = 91) *n* (%)	SI (*n* = 123) *n* (%)	*p*-Value
T/T	83 (91.2%)	111 (90.2%)	*p* > 0.05
T/C	8 (8.8%)	12 (9.8%)	
T **(wild allele)**	174 (95.60%)	234 (95.12%)	*p* > 0.05
C	8 (4.4%)	12 (4.88%)	

Caption: AS = Asymptomatic Group; SI = Symptomatic Group.

**Table 6:** Comparative genotyping of this cohort of professionals for the analyzed *TLR2* SNP related to COVID-19 severity.

Genotyping *n* (%)	AS (*n* = 91) *n* (%)	SCP (*n* = 35) *n* (%)	SSP (*n* = 08) *n* (%)	*p*-Value (AS vs. SCP)	*p*-Value (AS vs. SSP)	*p*-Value (SCP vs. SSP)
T/T	83 (91.2%)	31 (88.6%)	8 (100%)	*p* > 0.05	*p* > 0.05	*p* > 0.05
T/C	8 (8.8%)	4 (11.4%)	0			
T **(wild allele)**	174 (95.6%)	66 (94.3%)	16 (100%)	*p* > 0.05	*p* > 0.05	*p* > 0.05
C	8 (4.4%)	4 (5.7%)	0			

Caption: AS = Asymptomatic Group; SCP = Symptomatic Group with Pulmonary Compromise; SSP = Symptomatic Without Pulmonary Compromise.

## Data Availability

The original contributions to this study are included in the article. Further inquiries can be directed to the corresponding authors.
